# Reinforcing Feedbacks for Sustainable Implementation of Rural Drinking-Water Treatment Technology

**DOI:** 10.1021/acsestwater.3c00779

**Published:** 2024-03-26

**Authors:** Merel Laauwen, Saskia Nowicki

**Affiliations:** † School of Geography and the Environment, University of Oxford, South Parks Road, Oxford OX1 3QY, U.K.

**Keywords:** sustainable development, drinking-water safety, safely managed water supply, passive chlorination, UV disinfection, systems thinking, implementation science

## Abstract

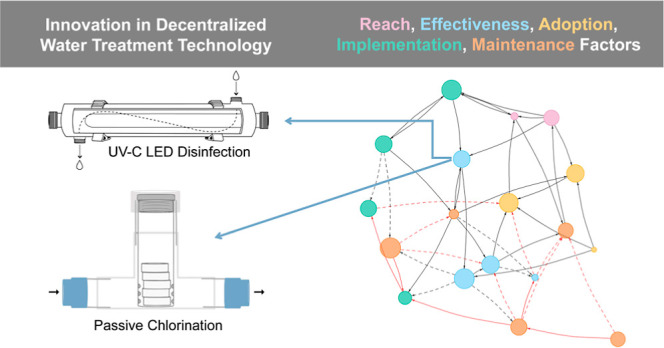

Progress toward universal access to safe drinking water depends on rural water service delivery models that incorporate water safety management. Water supplies of all types have high rates of fecal contamination unless water safety risks are actively managed through water source protection, treatment, distribution, and storage. Recognizing the role of treatment within this broader risk-based framework, this study focuses on the implementation of passive chlorination and ultraviolet (UV) disinfection technologies in rural settings. These technologies can reduce the health risk from microbiological contaminants in drinking water; however, technology-focused treatment interventions have had limited sustainability in rural settings. This study examines the requirements for sustainable implementation of rural water treatment through qualitative content analysis of 26 key informant interviews, representing passive chlorination and UV disinfection projects in rural areas in South America, Africa, and Asia. The analysis is aligned with the RE-AIM framework and delivers insight into 18 principal enablers and barriers to rural water treatment sustainability. Analysis of the interrelationships among these factors identifies leverage points and encourages fit-for-purpose intervention design reinforced by collaboration between facilitating actors through hybrid service delivery models. Further work should prioritize health impact evidence, water quality reporting guidance, and technological capabilities that optimize trade-offs in fit-for-purpose treatment design.

## Abstract 2

Improvements in rural water safety management are needed to achieve universal access to safe drinking water. This study identifies leverage points to sustain rural water treatment implementation.

## 1 Introduction

Inadequate access to safe drinking water leads to serious health impacts and deepens social and economic inequalities.^1,2^ Access to safe drinking-water is essential to human health and it is a basic human right.^3,4^ However, in 2020, one in four people lacked access to safely managed drinking water, and eight out of ten people who lacked even basic drinking water services were living in rural areas.^5^ Rural coverage of safely managed water services is lower than urban coverage in all of the United Nations Sustainable Development Goals (SDGs) country grouping regions.^5^ Microbial contaminants from human or animal feces are the primary threat to drinking water safety.^3^ Piped water supplies, boreholes, rainwater collection systems, and protected wells and springs (“improved” water supply infrastructure as defined for the Millennium Development Goals) have high rates of fecal contamination unless water safety risks are actively managed.^6^ Human and animal feces can spread pathogenic bacteria, viruses, protozoa, and helminths; therefore, to ensure the safety of drinking water, a series of barriers to fecal contamination should be implemented.^3^ The World Health Organization (WHO) recommends a risk-based approach that considers the need for water source protection, appropriate selection of water treatment technologies, and sound management of water distribution.^3^

Within the broader risk-based framing of water safety management, technological innovations may offer promising avenues to improve water safety. However, across the water, sanitation and hygiene (WASH) sector, a growing body of evidence shows that the expected improvements in health outcomes from technology-focused interventions are often elusive.^7−17^ This is due to the complexity of the links between WASH and health and also due to challenges of sustaining adherence to interventions.^18,19^ Here we focus specifically on drinking water treatment interventions. To increase drinking water safety in rural areas, decentralized water treatment approaches generally focus on either the household level [point-of-use, (PoU)] or the water-supply level [prior to the point-of-collection, (PoC)].

Household-level water treatment, including the use of filters, solar disinfection, boiling, or chlorination, has been widely promoted for years.^20^ While technologically effective, high adherence to PoU water treatment is required for positive health impacts to be realized.^21,22^ Yet, household-level water treatment methods have low uptake and sustainability in many contexts.^17,21^ Focusing on the household as the locus of responsibility for ensuring safe drinking water places an additional burden on individuals, often women, who may be limited in their ability to take-on this burden by poverty, gender norms, and the need to balance their effort across multiple priorities.^2,23−25^

Transformative advancements are needed in the rural water sector to develop service delivery models that shift the burden of water treatment away from households.^23^ Applying systems thinking to examine the challenges of safe water service delivery provides insight for the design of such models.^26,27^ In this study, we use an implementation science framework to explore a complex adaptive system (CAS) in which supply level rural water treatment interventions are embedded. We focus specifically on the implementation of passive chlorination and ultraviolet-C light-emitting diode (UV–C LED) disinfection. These two disinfection approaches were chosen due to their widespread uptake and technological advancements in recent years.^28−32^ Both approaches reduce health risk from microbiological contaminants in drinking water; both require ongoing maintenance by a local party; both are installed in-line so that they act on water flowing through a pipe, tap, or pump, either at the PoC or upstream;^33−35^ and both may require pretreatment filtration steps to be implemented if water has high turbidity (which reduces the disinfection efficiency for both UV and chlorine^36−38^). Beyond these commonalities, the two approaches have several important differences.

Passive chlorination is a form of water treatment that continuously and automatically doses chlorine, while operating without electricity (although electricity is intermittently required for on-site chlorine generation if adequate chlorine supply chains are not accessible). There are commercially available passive chlorination devices (e.g., Aquatabs Flo, Aquatabs Inline), or basic designs can be constructed from common materials that are used for building and maintaining small piped water schemes (e.g., AkvoTur, T-shaped chlorinator, pot chlorinator). Passive chlorination technologies vary widely,^39^ and factors that differentiate chlorine technologies include the form of chlorine used, cost, maintenance requirements, and compatibility with water supply infrastructure parameters like pipe sizes and flow rates.^39−41^ The mechanisms and advantages of different types of passive chlorinators were explored by Dössegger et al.^34^ Passive chlorinators are operated at the water supply level, but they can provide ongoing protection at the household level if dosing is sufficient to have adequate residual chlorine concentration.^34,39,42^ This is an important advantage of passive chlorination over UV-based disinfection, which provides no residual protection. Using chlorine for disinfection also has several disadvantages. Chlorine has varying levels of effectiveness against different microorganisms;^43^ it can be ineffective against hardy protozoa like cryptosporidium^44^ or some viruses.^45^ Implementation challenges can arise if water users object to chlorination because of changes in water taste/odor, for cultural or religious reasons,^46^ or due to concerns about disinfection byproducts (DBPs) that form when chlorine reacts with organic matter.^47^

Ultraviolet (UV) irradiation is capable of inactivating a broad spectrum of microorganisms without the use of chemical consumables (therefore, without taste/odor or DBP concerns). With UV, the mechanism of disinfection is agnostic to the taxa of the microorganism.^32^ However, microorganisms do have varying susceptibilities to UV irradiation: for example, cryptosporidium is easily inactivated but viruses are more difficult to inactivate.^48,49^ With treatment at high UV fluences, microorganisms are inactivated through the absorption of UV photons by proteins in the outer cell membranes, leading to disruption and consequent death of the cell.^50^ At lower fluences, microorganisms can no longer cause infection as the ability to replicate is disrupted.^50^ UV–C LED technologies are advancing at an unprecedented speed. Lui et al. found, in 2016, that commercially available UV–C LEDs were already technically effective in inactivating *Escherichia coli* and *Enterococcus faecalis*, and offered advantages in terms of speed and energy demand.^28^ Simons et al. calculated a 39% compound annual growth rate in commercial single-chip LED output power between 2005 and 2022.^51^ While conventional mercury-based bulbs require a warm-up time and thus must be in continual operation, LEDs may remain in low-power standby mode and only need to be engaged on-demand.^51^ The rapid advancement of LED efficiencies suggests that UV–C LED disinfection can open-up opportunities for more affordable, effective water treatment.^51^ However, the UV–C LED approach also has important disadvantages to consider. Unlike passive chlorination, UV-based disinfection relies on energy access, which is not reliable or affordable in many resource-constrained rural settings. The advancement of solar power technologies may alleviate this issue,^28,29^ but UV–C LED disinfection is still complicated compared to passive chlorination, particularly with regards to accessing specialized spare-parts.^28^ For example, supply chain issues related to microchip acquisition persist globally due to the dependence on several countries to coordinate materials and manufacturing.^52^

Current research priorities for UV-based water disinfection center on improving inactivation levels of microbial contaminants^53−55^ and developing technologies that are compatible with decentralized energy supply.^28^ However, implementation of UV–C LEDs in full-scale centralized or decentralized water treatment systems remains poorly characterized and understood.^56^ For passive chlorination, a 2022 critical review^30^ outlined key components needed for scalability: electricity access for on-site chlorine generation,^53,57^ residual disinfection, consistent water supply,^58^ low user burden,^59^ local manufacturing capacity, and affordable cost of technology and operations and maintenance (O&M). The review identified four research priorities including (i) strengthening supply chains, (ii) context-specific financial sustainability, (iii) remote monitoring and sensors, and (iv) handpump-compatible passive chlorinators.^30^

This study aligns with the research priorities for both UV–C LED and passive chlorination treatment approaches by using an implementation science framework. This framework guides our exploration of the barriers and facilitators for sustainable implementation of supply level water treatment in rural, resource-constrained settings. It enables development of a rich comparison of the implementation environment for passive chlorination compared to UV–C LED technologies.

## 2 Methods

Implementation science focuses on the gap between efficacy studies and real-world interventions at scale.^60,61^ Research examining the factors and processes that make WASH or other environmental health interventions successful is included within this scope.^27,62^ Intervention implementation studies use varying and overlapping theoretical, analytical and experimental methods that also draw on the aligned fields of translational research and systems science,^26,61,63−65^ with some key successes in improved health outcomes.^66−68^ In this study, we use an implementation science conceptual framework and system mapping method to guide and analyze key informant interviews. Our approach is an application of systems thinking, where “system” refers to the components and dynamic interactions of local context, management, supply chains, and other factors that collectively constitute the CAS of rural water supply.^26^ Thus, this study aims to understand the complexity of water treatment interventions beyond technical components. To avoid confusion, physical water supply and treatment “systems” are referred to as “technologies” or “infrastructure”.

### 2.1 An Implementation Science Framing

**Figure 1:**
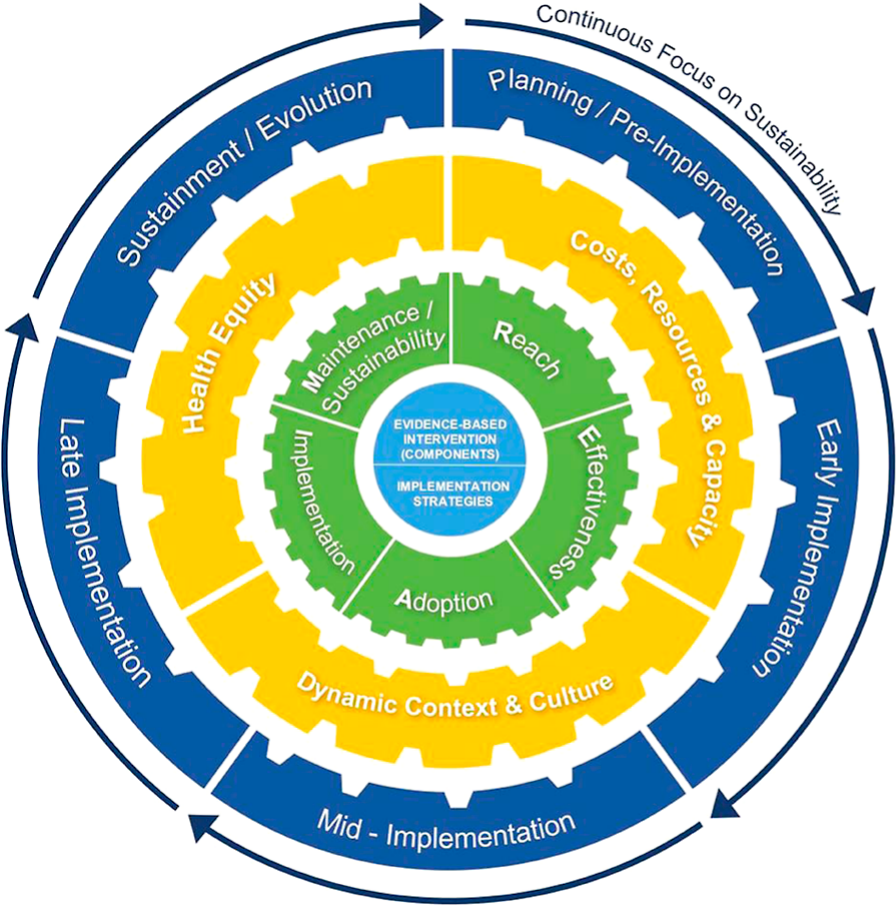
Updated RE-AIM Framework as presented by Shelton, Chambers and Glasgow 2020 [CC BY].^74^

The RE-AIM framework evaluates public health interventions according to five key dimensions: Reach, Effectiveness, Adoption, Implementation, and Maintenance/Sustainability, with long-term sustainability and health equity concerns having been added recently^69−74^ (Figure 1). The Reach dimension directs focus to the intervention target population and factors, such as user/customer profiles and collaboration between actors. It is most relevant during the planning stages of an intervention and is heavily influenced by costs, resources, and capacity considerations. The Effectiveness dimension includes consideration of factors that impact how well an intervention achieves the desired outcomes when implemented according to guidelines and protocols in the real world (as opposed to how efficacious it may be under controlled circumstances). This is a priority during the early stages of implementing an intervention. The Adoption dimension is about communication with the actors that are the intended adopters of the intervention and working to understand their perception of the intervention. It is heavily influenced by context and culture and dominates focus in the midimplementation phase. The Implementation dimension evaluates the extent to which the intervention is carried forward as intended, prompting considerations of financing, person-power, and roles and responsibilities. Within the framework, it is positioned for primary focus during the late implementation stage and is influenced by context, culture, and healthy equity considerations. Finally, the maintenance/sustainability dimension prompts consideration of long-term monitoring, stability of implementation models, climate resilience, and supply chains. This sustainment phase of an intervention is oriented toward advancing health equity, where all members of a population have fair opportunity for good health and well-being through a reduction of health risks that negatively impact marginalized groups and through provision of protective measures and care that is accessible to all.^74,75^ All five dimensions are intended to be explored within a broader and continuous focus on sustainability through all stages of an intervention, from planning to sustainment.

The initial development of the RE-AIM framework sought to improve public health interventions by promoting more efficient use of resources and by aligning different stages of development.^73^ More than 20 years later, the framework was adapted to include the longer-term perspective,^74^ which reflects a growing recognition of the dynamic, complex, systemic nature of public health interventions. Thus, RE-AIM provides a useful framing for our investigation of rural water treatment by positioning treatment technologies within the complex systems that they aim to change.^74^ RE-AIM is also preferred because it substantially prioritizes sustainability and health equity, which receive more limited consideration in many other implementation science frameworks and theories.^69−71,76^

### 2.2 Key Informant Interviews

Semistructured interviews were conducted in June and July 2022 with key informants who work in academia, NGOs, commercial companies, research institutes, public health institutes, or a combination (Table 1). An interview guide was developed based on the components of the RE-AIM framework and interviewees were asked about general facilitators and barriers to implementation, water user perceptions, communication, management and financing structures, supply chains, health impacts, long-term resilience, and key future improvements. Interviews were conducted via Zoom and lasted between 26 and 105 min.

**Table 1:** Summary of Key Informant Characteristics

actor category	technology		role		country of organization		country of operations	
	passive chlorination	UV–C LED disinfection	operational	managerial	low- or middle- income country	high income country	low- or middle- income country	high income country
academia	5	6	0	11	0	11	8	2
academia and NGO involvement	2	1	1	2	0	3	4	0
NGO	6	1	4	3	3	4	9	0
research institute	3	0	0	3	0	3	5	0
public health institute	2	0	0	2	0	2	1	1
total	18	8	5	21	3	23	27	3

The main criteria for inclusion as a key informant was involvement in at least one project to implement passive chlorination or UV–C LED water disinfection technologies in rural areas of low- or lower-middle-income countries. Academic and research institute actors were included because a large portion of the ongoing work on passive chlorination and UV–C LED technology implementation is experimental; thus, researchers are heavily involved. Eighteen key informants were experts in passive chlorination and 8 in UV–C LED technologies. They work on treatment implementation projects that deal with a range of technology maturity levels including established/mass manufactured devices, “build-your-own” devices, and novel innovative design development. Three projects were identified initially through the authors’ existing networks from previous research on rural drinking water safety in Kenya, Nepal, Guatemala, Honduras, and Nicaragua. An additional 13 projects from rural areas in Australia, Bangladesh, Japan, Philippines, Vietnam, Haiti, United States, Uganda, Malawi, Tanzania, and Ghana were identified through snowball sampling.^77^ In 7 cases, multiple key informants from the same project were interviewed to comprehensively cover activities pertaining to all five dimensions of the RE-AIM framework. Snowballing continued until new thematic content plateaued^78^ and referrals for key informants from different projects stalled, with a total of 26 interviews completed.

The broad geographical scope of this study was suitable for the exploratory nature of the research, particularly due to the narrow technological scope. Of the 26 key informants, 17 work at either an academic institution, have both academic and NGO roles, or work at a research institute. These key informants provide a broad perspective of the systems analyzed in this study because they have consolidated sets of experiences through multiple case studies over many years. This broad perspective is valuable for developing systems maps that are more generalizable across different contexts. However, the use of this broad perspective has several important limitations. First, the perspective of implementers with a more direct and highly context-specific implementation experience is underrepresented. Second, research on passive chlorination and UV–C LED technologies has predominantly been led by institutions in high-income countries, so 88% of the key informants are based in high-income countries despite 90% of the water treatment implementation projects that they spoke about being in low- or middle-income countries. Third, the key informants of this study are at least one degree removed from water users, limiting a comprehensive understanding of the entire network of actors involved in water treatment implementation. In future work, a more granular geographic focus with better representation of implementers and potentially water users would allow for more detailed, context-specific mapping of decentralized water treatment implementation.

The University of Oxford Central University Research Ethics Committee (CUREC) approved the research based on the study’s CUREC application (SOGE1A2021–031).

### 2.3 Content Analysis and Systems Mapping

Audio recordings of the interviews were transcribed verbatim using Trint transcription software, followed by manual correction. The transcripts were then analyzed following the qualitative content analysis method described by Drisko and Maschi, 2015.^79^ All transcripts were initially coded to a node framework structured according to the five dimensions of the RE-AIM framework: Reach, Effectiveness, Adoption, Implementation, and Maintenance/Sustainability, which were further separated into subnodes to classify facilitators and barriers to successful implementation of passive chlorination and UV–C LED water treatment technologies. In this way, the five dimensions of the framework were used as the foundation for the “open coding” component of the qualitative content analysis.^79^ To synthesize the coding results, a conceptual map was produced to represent the breadth of implementation factors organized into categories corresponding to the RE-AIM framework (Supporting Information Figure 1). These factors were summarized into principal topics that encompass enablers or barriers to sustainable rural water treatment (Table 2). To give a sense of the extent to which topics were explored in the interviews, Table 2 reports (i) the number of interviews in which the topic was discussed and (ii) the percentage of those interviews in which the topic was discussed, i.e., the coding coverage expressed as average and range.

**Table 2:** Summary of Principal Enablers of and Barriers to Sustaining Rural Water Treatment, Identified From 26 Key Informant Interviews

topic	description	quote(s)	interviews	% coding coverage
REACH				
R1 user/customer base (enabler)	The customers of UV and passive chlorination technologies range from NGOs, private companies, and local governments to individual homeowners. In most settings, these technologies are installed to serve small communities, schools, or healthcare facilities. The profile and proportion of populations reached (and potentially reached) by interventions was identified as a determinant of their scope for impact and whether they can advance health equity, and was mentioned explicitly as an enabler in a subset of the interviews.	“The customers could be NGOs that are partnering with healthcare facilities or the local government that is providing water at schools and other institutions. It could be individual homeowners. It could be private. The device itself could work in a lot of settings. So, I think it’s going to be kind of a wide range of customers.” (Cl_04)	5	1.5 (0.9–2.3)
R2 collaboration between facilitating actors (enabler)	Various actors, including manufacturing companies, research organizations, service providers, NGOs, government, regulators, donors, and investors have actual or potential roles in facilitating rural water treatment implementation. Collaboration and information sharing between these facilitating actors was deemed as important or essential in many interviews: the nature of this collaboration influences the profile and proportion of populations that are reached by interventions, and has impacts on technology development and the sustainability of financing.	“I feel like there’s a couple of bad eggs that like are not about collaboration. It’s all about touting their best design and their best approach. But a lot of us on the other side, we’re connected··· You know, we love information, we have a lot to share, but we learn as well.” (Cl_18)	19	3.4 (0.4–9.7)
EFFECTIVENESS				
E1 dosing/fluence calibration and reliability (enabler)	For both UV disinfection and chlorination, correct fluence/dosing is a core performance issue. The capacity to adapt to variability in water flow rates and water quality is important in most rural systems, which are often characterized by intermittent flow and minimal or no pretreatment stages before disinfection. Dosing/fluence must be designed to balance efficient use of resources, organoleptic issues, disinfection sufficiency, and recontamination risks.	“I think one of the biggest barriers we have is the difficulty of ensuring a consistent chlorine dose with inconsistent flows of water.” (Cl_05)	22	5.2 (0.6–17.1)
E2 fit-for-purpose technology design (enabler)	In the past decade, many new decentralised water treatment technology options have been developed. These are intended for use in a wide range of settings. There are trade-offs to consider when selecting between a chlorination or UV treatment approach, when selecting particular design options within these approaches, and when deciding whether and how to include source protection and predisinfection steps.	“You can’t say inline chlorinator A is going to be the same as B, they could be completely different products. So, I think understanding products that provide more consistent quality dose is really crucial. . . Inline chlorinators have a place. . . but that place is not the entire world.” (Cl_05)	24	9.4 (1.0–24.1)
E3 disinfection byproduct (DBP) risk (barrier)	DBPs present a potential health risk and associated acceptability risk for chlorination interventions. The evidence of DBP health impacts is inconclusive, but there are also regulatory requirements and water user acceptability factors to consider. Variability in the concentration of organic matter in pretreatment water makes DBP management more complex.	“It should be really clear to mention that the risk of these byproducts, as compared to the risk of infectious disease, is just so much lower that we shouldn’t avoid chlorine for fear of exposure to various chemicals.” (Cl_07)	3	2.0 (0.8–2.9)
E4 technology development (enabler)	Key areas of decentralized water treatment technology development relate to energy consumption and material characteristics such as recyclability, lifetime, and availability. For example, the cost of UV–C LED bulbs has reduced 100-fold in the past decade, driving rapid development of UV–C LED technologies, which have become more electrically efficient, more powerful, cheaper, longer-lasting, more compact, and easier to operate. Water quality sensor technology is also advancing with important implications for monitoring capabilities.	“LEDs have sparked interest, and I think that’s spurred more interest in implementation and also advances in solar power as an option for powering UV systems.” (UV_04)	17	5.8 (1.1–20.7)
ADOPTION				
A1 water user acceptance of water treatment (enabler)	Acceptance of treatment interventions is influenced by education, prior experience, local norms, information dissemination and other factors. Water users that are closer to the point of chlorination receive water with higher chlorine residual concentration and may have more taste/odor complaints. Besides organoleptic aspects, knowledge about disinfection varies and myths, suspicions, and religious positioning can impact acceptance. Labour and cost are also key factors: treatment implemented within the water supply prior to point of collection has advantages over household-level treatment because of economies of scale and no household labor requirement.	“If people are unhappy with you putting the chlorine, you know, forcing them to get chlorine, they can switch sources. They can go to a very polluted source because they dislike your chlorine. And myths about chlorine are very prevalent in rural areas. In Uganda, for example, it is common to believe that it leads to fertility issues.” (Cl_11)	25	6.5 (0.3–12.4)
A2 communication with water users (enabler)	Communication with water users is perceived to be beneficial for water treatment interventions. Differences in how engagement is done and what is communicated are important. Water users must know that chlorination or UV disinfection is occurring, and there must be space for them to ask questions or express concerns. There are mixed opinions on whether researchers or other foreign actors should have direct contact with water users. Language and cultural barriers may hinder effective communication, and in such settings in-country collaborators or uptake partners should take the lead.	“I’m very adverse to having some outsider come tell you what you need. So, if you can have people established in that community, working alongside community members, that’s the only way, the best way, to engage and exchange knowledge on those types of opportunities.” (Cl_14)	24	4.9 (0.3–9.1)
A3 participatory planning (enabler)	The involvement of water users and other local actors in the implementation process is an important factor in implementation adoption. Key informants emphasized the importance of collaboration with local institutions. Roundtable discussions and community meetings for information dissemination are common, but codesign and participatory planning is uncommon and is considered to have good potential to improve the design and acceptability of treatment interventions.	“I feel like really starting with engaging the community you’ve actually impacted and using participatory research methods where you are actively conducting needs assessments or asking the community to work on the research in terms of designing experimental studies or the actual implementation plan.” (Cl_04)	4	1.0 (0.3–2.2)
IMPLEMENTATION				
I1 long-term financing (enabler)	Sustained long-term financing is required to enable ongoing operations and maintenance of rural water treatment. Securing initial and ongoing sources of financing is a key challenge for technology implementation efforts.	“I mean, I hate to keep repeating myself, but I think it really sort of comes back to the education, economic issues and business issues. I think those are really the biggest barriers.” (UV_09)	25	8.2 (0.9–33.4)
I2 burden on households and community (barrier)	The distribution of the labor and other operating costs that are required to sustain water treatment is a key consideration. Where this labor is distributed to households, as with household-level water treatment technologies, or to community water management committees that have no external support, it represents a burden, often borne by women and girls, that has been found to be widely untenable in impoverished communities.	“Whether you’re talking about household, community, institutional utility, if you assume that the users are going to take ownership and maintain the system on their own, you might be ignoring a large part of the financial and time and cost burden which are often gendered, that are associated with this long-term operation and maintenance.” (Cl_04)	19	4.4 (0.9–16.2)
I3 costs of installation and operations (barrier)	The costs of chlorination and UV technology products, including consumables, spare parts, maintenance, and monitoring costs are a consistent challenge for projects that are focused on rural areas of LICs and LMICs. Introducing new costs in resource constrained settings is challenging and projects are looking to fit-for-purpose technology design and innovative service delivery models for ways forward.	“I think that’s the biggest challenge, really, is if you want to make it financially self-sustainable. How do you do that? People can’t afford it basically. Or if it’s not priority for people’s money.” (Cl_01)	19	1.9 (0.5–5.9)
I4 supported service delivery (enabler)	Operation and maintenance (O&M) services must be in place to ensure that treatment technologies remain functional after installation. Water committees or boards, consisting of local resident members who are responsible for operating the technologies through nonpaid positions, are widely cited as having a role in O&M. However, hybrid institutional arrangements are sought to support communities with technical capabilities, supply chains, and long-term financing. Interviewees emphasize the importance of engaging with the “right” partners, stating that these should either be local entities or actors that will work locally for a long time.	“You really need some sort of outside organization support for the maintenance of these chlorination technologies or, you know, whether it’s just having some sort of technical support, like to consult if something goes wrong to help, you know, maintain the supply chain, get replacement parts, either some local NGO or just as much more buy in from local governments is something that I think is going to be required.” (Cl_02)	17	5.5 (0.4–20.1)
MAINTENANCE/SUSTAINABILITY				
M1 evidence of improvement in health outcomes (enabler)	Ultimately, the purpose of drinking-water treatment is to reduce health risk and improve health outcomes. Interviewees discussed the challenges of measuring the health impacts of treatment implementation. Understanding and evidence of health impacts is important to sustain the justification for treatment and motivation to collaborate on development and implementation of treatment technologies.	“When it comes to measurable health impacts, a lot of the big studies that look at various interventions are showing that water is quite a small piece of that puzzle. It has a lot to do with malnutrition, also with hand hygiene.” (Cl_07)	16	3.7 (1.4–8.1)
M2 supply chain challenges (barrier)	For chlorine and UV treatment, as well as water quality monitoring, nascent or nonexistent markets and the remoteness of rural localities creates challenges for the consistency and affordability of access to components and consumables. These supply chain issues are a key consideration for fit-for-purpose design and a key limitation on technology selection.	“We, let’s say, pushed to most (to choose) the locally, locally built chlorinators, I mean the parts are quite easily available. Sometimes they had to go to the capital to get parts, but most of them were available on the local markets.” (Cl_08)	26	9.1 (0.6–17.6)
M3 complex pretreatment water quality (barrier)	UV radiation and chlorine are less effective when applied to turbid waters. High concentrations of dissolved organic matter react problematically with chlorine, increasing DBP risk. Furthermore, disinfection does not reduce risks from chemical contamination which must also be considered for full drinking water safety to be achieved. In cases where turbidity and water chemistry challenges are substantial, pretreatment may be warranted before disinfection with chlorine or UV.	“I think what would make sense if you have higher turbid water or other chemical or biological contaminants would be to pre-treat the water that is coming in.” (Cl_04)	18	4.7 (1.0–16.9)
M4 climate change and climate variability risks (barrier)	Climate change and climate variability influence the complexity and predictability of pretreatment water quality challenges. They are also associated with wider risks to WASH infrastructure from extreme events, particularly drought and flooding.	“So, you know, I think we’re gonna see more and more WASH related challenges with climate, flooding, for example, or drought. I mean, it’s weird because it’s kind of at the extremes.” (Cl_15)	18	3.0 (0.1–8.2)
M5 water quality monitoring (enabler)	Water quality monitoring is complementary to water treatment implementation, it is required to calibrate dosing/fluence, manage DBP risk, and to develop the design and operation of treatment technology to improve effectiveness. In 3 interviews, informants also discussed the role of water quality monitoring for identifying emerging contaminants that affect the complexity of pretreatment water quality.	“Climate change is a challenge because it changes the quality of our source waters. And that can make it harder to treat them. It can add new pathogens or new contaminants to the water that old UV systems are not designed to handle. And so, monitoring of source of water and monitoring of the performance of UV systems against those sorts of waters, I think will be important.” (UV_04)	6	2.3 (0.3–4.8)

**Figure 2:**
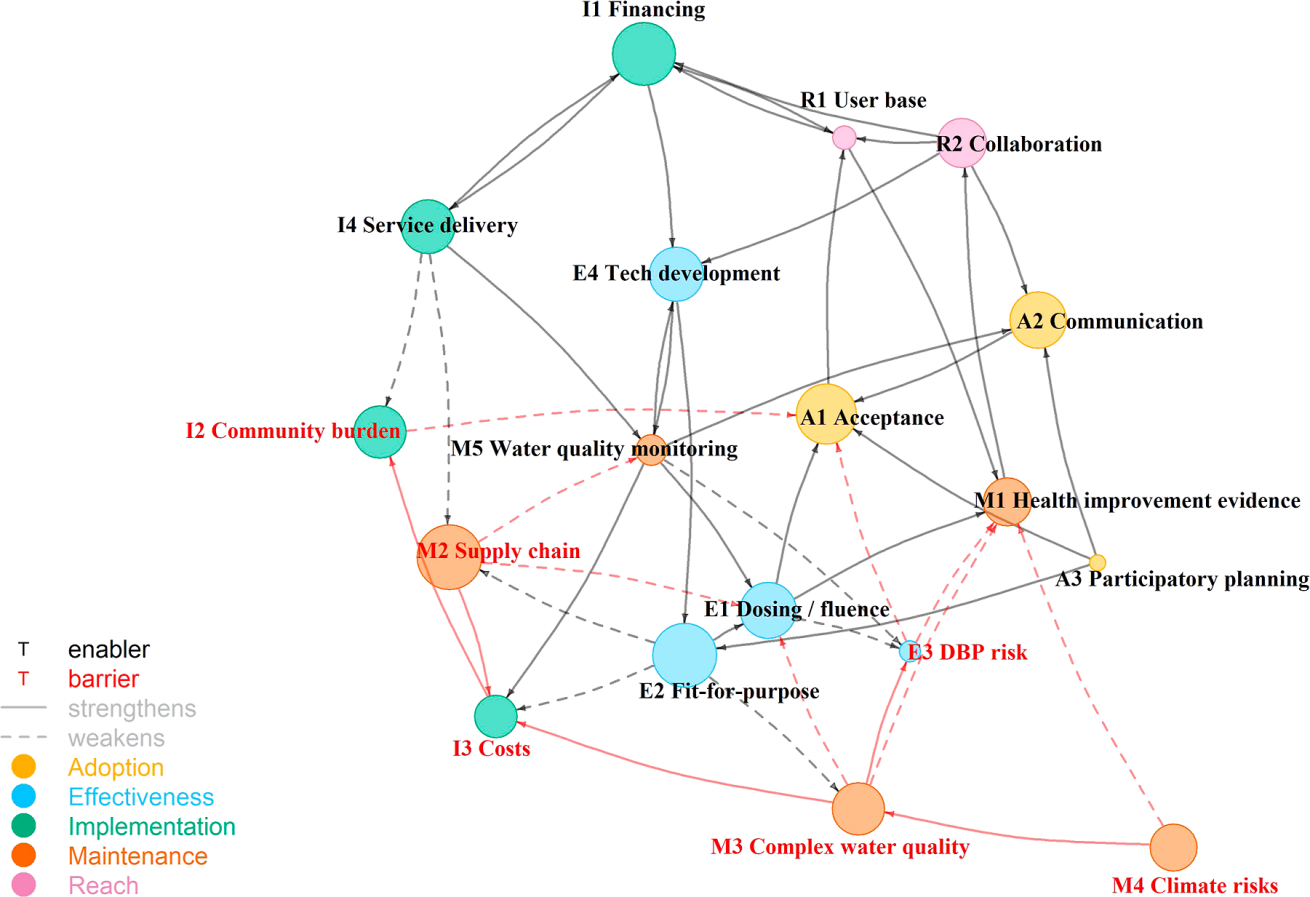
Network diagram showing relationships between the principal enablers and barriers to sustained rural water treatment. Each node is described in Table 2. The node size is the natural logarithm of the number of interviews that discussed each topic multiplied by the average coding coverage for that topic. Links between nodes are shown, where key informants discussed connections between the topics. The diagram layout is generated by the Fruchterman-Reingold force-directed layout algorithm in the R “igraph” package.

To further explore the systemic complexity of rural water treatment implementation, a network diagram was then produced by using a modified fuzzy cognitive mapping (FCM) technique to synthesize the key relationships between enablers and barriers (Figure 2). FCM is a form of systems mapping that uses nodes and edges to explore how causal influences propagate through a dynamic system.^80^ Traditionally, FCM includes assigning numerical values to both the nodes and edges in the system map to represent either confidence in or magnitude of causal effects.^80^ Given the qualitative nature of the data in our study and because we did not ask key informants to rank the importance of different enables and barriers, we did not make numerical assignments in this way. Instead, we assigned node sizes by calculating the natural logarithm of the number of interviews that discussed each topic multiplied by the average coding coverage for that topic. Links (edges) between topics (nodes) are shown where any key informant discussed causal connections between the topics, and we assigned edges as a binary of having either a strengthening or weakening influence. Thus, the network diagram in Figure 2 visualizes the collective response from our key informants, with node size roughly representing the depth of content for each topic. Figure 2 is intended to be used as a conceptual tool, to represent the complexity of rural water treatment implementation, and to generate discussion. It should be understood as a partial representation of an evolving CAS.

## 3 Results and Discussion

Content analysis of the 26 interviews conducted for this research produced a set of 110 factors that influence the implementation of rural water treatment with passive chlorination or UV technology. Organized by the dimensions of the RE-AIM framework: reach (12 factors), effectiveness (15), adoption (19), implementation (31), and maintenance/sustainability (33), these factors demonstrate the breadth of considerations for rural water treatment initiatives (Supporting Information Figure 1). The factors can be grouped into 18 principal enablers of or barriers to sustaining rural water treatment (Table 2). The label of enabler or barrier is allocated based on whether the presence of the factor in question is enabling or inhibiting. Thus, enablers are beneficial when they are present but can be barriers or have neutral influence in their absence. Barriers are problematic when they are present, but their absence can be neutral or enabling.

The enablers and barriers described in Table 2 are components of a CAS for rural water supply. Seven of the key informants spoke about technology implementation with explicit systems-language. For example, one key informant said:

*“There’s a lot of confounding variables, because it is very hard just to put this technology in and then walk away and let it run. It definitely has to be a systems approach for the technology to succeed at all. So then if there is a decrease in disease, it’s attributable to both the technology but also the system that has been created” (Cl_03).*

Most informants discussed technology implementation in more linear terms of siloed causes and effects. However, analysis of the relationships between the 18 principal enablers and barriers identified from the interviews provides insight into the propagation of causal influence within a system (Figure 2), which is key to understanding the dynamics of a CAS.

The demography and characteristics of a technology user/customer base determine whether interventions advance health equity or reinforce inequities. In 5 interviews, we were told that the potential user/customer base for decentralized water treatment technologies is vast and diverse (Table 2: R1). This is supported by a recent study that estimates 1.2 billion people living in rural areas globally are using microbially contaminated water sources that are compatible with passive chlorination treatment technology.^81^ Our key informants indicated that the two most prominent enablers of more extensive technology reach are collaboration between facilitating actors (R2) and water user acceptance of water treatment (A1). We examined how these enablers are reinforced or undermined.

### 3.1 Evidence of Health Improvements Catalyzes Collaboration Around a Shared Purpose

The intended purpose of rural water treatment is to sustain improvements in health. In keeping with this positioning, a key principal enabler in the maintenance/sustainability category of our results is measurable improvement in health outcomes (Table 2: M1). Evidence and perception of positive health impacts were found to promote collaboration between facilitating actors (R2), particularly between funders and implementers. For example, in July 2022, GiveWell recommended an Open Philanthropy grant of up to 5.6 million USD to fund an in-line chlorination program in Malawi.^82^ Their recommendation relied on an evaluation of cost-effectiveness in improving health outcomes and emphasized the value of high-quality data to improve the precision of cost-effectiveness estimates. Health impact evidence can thus support funding partnerships that enable water treatment interventions to have a more extensive reach (R1), including reaching people who are vulnerable in marginalized contexts. Thus, a reinforcing feedback is described where evidence of health improvements (M1) reinforces collaboration (R2) and financing (I1), which increases the scale of the user base (R1) and reinforces health improvements and the potential to provide evidence of health improvements (M1).

Our key informants pointed to water quality complexity (M3), climate risks (M4), and DBP risk (E3) as influences that weaken or contravene the usefulness of health improvement evidence (M1). More specifically, it was acknowledged that the complexity of confounding factors makes it difficult and expensive to evaluate health outcomes from drinking-water treatment interventions. Research has shown that the presence of passive chlorinators in rural communities resulted in significantly improved water quality,^2^ but few studies have linked to health outcomes. Our key informants discussed perceived health improvements based on anecdotal evidence of reduced gastrointestinal symptoms among water users, and there is some systematic evidence of impact–for example, a randomized controlled trial in Dhaka showed evidence of a reduction in childhood diarrheal disease due to passive chlorination interventions.^31^ Overall, however, this is an area for further work that could be used to leverage funding for rural water treatment implementation.

### 3.2 Communication and Service Delivery Approaches Drive Acceptability

Acceptance of a water treatment intervention by water users (Table 2: A1) is an adoption-related enabler that was discussed in all but one of the interviews as a major determinant of the user/customer base (R1). Specifically, acceptability of taste, odor and perceived risk is important to ensure continued consumption or willingness to pay for treated water sources, indicating the need for well-calibrated, reliable dosing (E1) and site-specific acceptability research.^30,46^ Collaboration between facilitating actors (R2) has a role here in facilitating access, resources, and appropriate messaging for communication with communities (A2). Interviewees said that the results from water quality monitoring, another enabler (M5), can be used to communicate the benefits of water treatment to water users. However, uncertainty in interpretations of water-related health risk and the relative sparsity of rural water quality monitoring means that communicating water quality results can be difficult and there is limited precedent to draw upon.^83^ Reporting approaches need to contextualize water quality results to mitigate the risk of maladaptive cognitive, behavioral, and institutional response outcomes.^24,83^

Beyond communication efforts, in 4 cases, interviewees also spoke about the value of engaging with communities and local institutions in participatory planning processes (A3). A key consideration for planning implementation of water treatment is the distribution of costs (I3). Costs, including labor, reduce water users’ acceptance of treatment implementation (A1) when they are borne by the community (I2). In contexts of poverty, by definition, people are vulnerable, and meeting basic needs is a daily struggle. Allocation of water treatment costs exclusively to community-level is problematic for the same reasons that have been extensively explicated for the widespread failure of unsupported community-based management to sustain water supply functionality.^84,85^ In developing beyond the community-based management model, the rural water sector is experiencing rapid innovation and proliferation of service delivery models. Seventeen key informants discussed this topic, highlighting the need for hybrid institutional arrangements to sustain the operation and maintenance (O&M) of treatment technologies (I4). Hybrid service delivery approaches were noted as a means to consolidate operating costs and labor that would otherwise be borne by households or communities alone (I2), facilitate water quality monitoring (M5), buffer against supply chain volatility (M2), and leverage multiple funding sources for long-term financing (I1).

Key informants spoke about challenges with introducing additional cost to water service delivery in resource constrained settings (I3). Most of the focus was on operating costs, recognizing that capital expenditure for small-scale, decentralized water treatment is typically low relative to the overall capital expenditure for water supply systems. All but one key informant raised the importance of long-term financing (I1). Specific funding arrangements and service delivery models vary by the context. Based on our interviews, external funding has most commonly been acquired through research grants and development project funding from foundations, international development organizations, and government development funding. Several of the professionalized water service delivery organizations that we engaged with in this research are trialing results-based funding arrangements to encourage further investment in rural water service provision. Professionalized service delivery (I4) and long-term financing (I1) were discussed as mutually reinforcing enablers of rural water treatment. Additionally, commercial entities, such as manufacturers, were found to collaborate on projects by donating devices or refills in-kind. Two key informants also spoke about the funding potential from carbon credit schemes. Since passive chlorination is an alternative to boiling water, which is done by burning wood, there is an indirect reduction in carbon emissions; however, carbon credits are not a predictable or stable source of finance as regulations change on a yearly basis and the verification process can take several years.

### 3.3 Fit-For-Purpose Technology Mitigates Implementation and Maintenance Barriers

In the previous sections, collaboration of facilitating actors (R2) is discussed primarily in relation to financing (I1) and a technology’s user/customer base (R1). Actor collaboration also has an important influence on technology development (E4). There remains a division between research and practice within drinking water treatment. Research, and the experimentation involved are essential for technology development (Table 2: E4), but research timelines and funding are often limited to only a few years. NGOs and other implementers, on the other hand, seek to roll out programs that will have benefits for as many people as possible, and thus may not have the capacity for rigorous data collection and experimentation, which is very expensive. To bridge this gap, key informants highlighted a need for more collaboration between facilitating actors (R2), particularly knowledge exchange across research, implementation, and commercial spaces.

A key objective of collaborative technology development is to improve fit-for-purpose design (E2). “Fit-for-purpose” simply means that something does what it is intended to do. The challenge is to design treatment technologies that are fit-for-purpose in different contexts. Interviewees emphasized that the effectiveness of water treatment technologies in a particular context is determined by their appropriateness for the water supply infrastructure and pretreatment water quality. Fit-for-purpose technology design is strongly linked to two principal topics: costs of installation, O&M (I3) and dosing/fluence calibration and reliability (E1), which is further linked to management of DBP risk (E3). Many design options exist,^8,34,86−89^ with a multitude of trade-offs to consider. However, there is still room for product design growth, particularly regarding trade-offs between precise dosing, device complexity, and affordability. There are also key trade-offs to consider when choosing between chlorination and UV disinfection approaches. Ongoing technology development (E4)—especially in UV–C LED capabilities–means that these trade-offs are evolving and fit-for-purpose design is improving. Two key fixed considerations, however, are that UV treatment is more effective against a broader range of pathogens,^54^ but provides no residual disinfection capacity, making it potentially best applied together with chlorination for a better overall performance.

Irrespective of treatment design, water quality monitoring provides critical information feedback to understand the effectiveness of disinfection (M5). Although this topic was only raised by 6 key informants, they highlighted links between water quality monitoring and many other nodes in our systems analysis, thus M5 has more links than any other topic (Figure 2). An increase in water quality monitoring leads to increased costs (I3), which (as a barrier to sustaining treatment and related activities) could lead to a subsequent roll-back of monitoring. However, this simple balancing feedback loop is made more complex because through several causal connections (Figure 2), monitoring can become a strengthening force for financing arrangements that offset the costs of doing the monitoring. This applies to the extent that information from monitoring is useful to improve the effectiveness, acceptability, and perceived value of treatment. Excessive monitoring should still be counteracted by the associated cost. Development in water quality monitoring capabilities is another area that can reinforce fit-for-purpose design (E2) to reduce costs (I3) while strengthening water users’ acceptance of water treatment (A1) through improved dosing (E1) and communication (A2). Research is ongoing in this space, focusing particularly on regulatory compliance, indicators for microbial activity, and sensor development.^28,30,90,91^

### 3.4 Leverage Points for Sustainable Rural Water Treatment

The results of this study demonstrate the systemic complexity of decentralized water treatment implementation in resource-constrained settings. Beyond the ability of a technology to improve the microbial safety of a water supply, the sustainability of treatment is dependent on a broad set of socio-economic and environmental factors. By distilling the principal enablers and barriers discussed in key informant interviews and by exploring their interrelationships, we identify multiple forms of leverage through which the sustainability of water treatment can be reinforced. In CAS, leverage points are processes or activities wherein a discrete intervention will influence wider system behavior toward an intended purpose.^92^ Meadows categorized 12 types of leverage points that scale from the positional leverage of mindsets/paradigms, which have the broadest influence but are most difficult to change, to the physical leverage of system component parameters, which are easier to change but have more localized influence.^92^ Our analysis, as laid out in the previous sections, identifies physical, feedback, and institutional forms of leverage for water treatment sustainability.

The results encourage an orientation toward fit-for-purpose technology design. Rather than looking for a “silver bullet” technology, the sector can develop a portfolio of treatment designs and implementation protocols that are suited to different environmental and institutional settings, even within a single service area. Technology development grapples with trade-offs between dosing effectiveness and device simplicity, with strong implications for health impacts, user acceptance, ease of O&M, supply chain reliability, and affordability. The specific form of these trade-offs varies extensively between contexts, so locally informed design and O&M decisions are warranted. Hybrid service delivery models are found to support the feasibility of a fit-for-purpose approach by (a) strengthening financing and monitoring and (b) mitigating challenges of distributing monetary and labor costs and buffering supply chain volatility. With reference to Meadows’ framework,^92^ this can be understood as localized leverage (from modification of physical events and infrastructure) that is reinforced and scaled by feedback and institutional leverage from efficient structuring of information flows, financing, and management.

At a structural level, collaboration between actors is found to influence the sustainability of rural water treatment through multiple pathways. Knowledge exchange on the development of technological capabilities, service delivery, and related financing models is a key area for cooperation that aligns with a fit-for-purpose approach. In contrast, seeking “silver-bullets” and market dominance can create a competitive orientation that hampers knowledge exchange. Improved communication will ensure a faster progression of best practices to make interventions more accessible and locally appropriate. Working groups, knowledge exchange platforms, and communities of practice are facilitating the sharing of innovation and best practice. The International Ultraviolet Association (IUVA), formed an SDG Task Force that meets regularly to share research and practitioners’ experiences. With similar intentions of networking learning, an online Community of Practice on Decentralized Chlorine Use has formed under the leadership of PATH and EOS International (see the Community terms of reference and contact details in Supporting Information Annex 1).

Beyond knowledge exchange between facilitating actors, engagement with water user communities is highlighted as a priority in the mapping of our results (Figure 2). The nodes representing communication with water users (A2) and water users’ acceptance of water treatment (A1) are among the largest in the map because they were discussed in more than 90% of the interviews with average coding coverages of 4.9 and 6.5%, respectively. In comparison, participatory planning (A3) was discussed only in 15% of interviews with an average coding coverage of 1%. Despite its relatively small size, the participatory planning node is situated as an enabler of communication, acceptance, and fit-for-purpose design–it may represent an overlooked area for strengthening that could leverage substantial improvement in the sustainability of treatment implementation.

## 4 Conclusions

In summary, taking a siloed approach to decentralized water treatment, solely through a technological lens, results in failed projects and stranded assets. To avoid this, systems-based analysis reveals the broader socioenvironmental factors and feedback loops that determine the sustainability of water treatment implementation. While this study focuses on passive chlorination and UV–C LED disinfection as its case studies, many of the identified enablers and barriers are generally applicable to other WASH interventions as well. Water treatment at the water-supply level (as opposed to household-level) is always an addition to an existing water service; therefore, an enabler or barrier to water services may also be an enabler or barrier to water treatment. The results of this study encourage a fit-for-purpose approach to intervention design that is reinforced and scaled through hybrid water service delivery models. Key information flows, financing, and management arrangements can be strengthened by collaboration among facilitating actors and with water users. Further attention is also encouraged to invest in and develop new forms of evidence of the health impacts and wider benefits of water treatment (Section 3.1), guidance for appropriate water quality reporting practices (Section 3.2), and technological capabilities that mitigate or help to optimally balance trade-offs in fit-for-purpose treatment design (Section 3.3).

## Supporting Information Available

Terms of reference of the community of practice on decentralized chlorine use (PDF)
Conceptual visualization of key categories and factors (PDF)
