# Supplementary material for: Reinforcing Feedbacks for Sustainable Implementation of Rural Drinking-Water Treatment Technology
Source: ACS ES T Water. 2024 Mar 26;4(4):1763–74. doi: 10.1021/acsestwater.3c00779 (PMC11019543; doi:10.1021/acsestwater.3c00779)
Supplement: Supplementary file 1 — ew3c00779_si_001.pdf [file ew3c00779_si_001.pdf]

Decentralized chlorine use for water treatment and infection prevention and control

A Global Community of Practice

Background

Infection in healthcare settings

Poor water, sanitation, and hygiene practices contribute to the spread of infections and negative health outcomes in healthcare facilities and communities. Healthcare acquired infections (HAIs) negatively affect hundreds of millions of individuals worldwide, namely mothers and infants. Patients in low-resource countries face an increased risk of exposure to HAIs: 3–20 times higher than patients in similar settings in high-income countries.[i] Bacteria account for roughly 90% of all nosocomial infections, with protozoa, fungi, viruses, and mycobacteria accounting for the remaining 10%.[ii] Factors contributing to this risk include unhygienic environments and lack of adequate handwashing infrastructure, and lead to disastrous outcomes: 10–16% of global maternal and newborn deaths are attributed to sepsis.[iii,iv] Nearly all of these deaths occurred in low-and middle-income countries.

Chlorine is a widely used, effective chemical disinfectant recommended for infection prevention and control in health care settings. However, despite its proven effectiveness, the lack of consistent chlorine availability limits the ability of healthcare facility staff to provide a safe and hygienic environment for patients. A global survey of healthcare facility conditions in 78 low-and middle-income countries revealed 36.4% lacked chlorine solution for disinfection.[v] A PATH chlorine inventory analysis across 18 HCFs in Ghana and Uganda found that healthcare facilities face an average of 1.6 to 2.5 stock outs of chlorine per year, totaling roughly 55 to 74 days per year in which HCFs did not have chlorine. Weak supply chains, burdensome procurement processes, and insufficient budgets contribute to an unsteady supply of chlorine in HCFs, a challenge further exacerbated during times of crisis.

Household and community drinking-water

Nearly 25% of the world's population as of 2020 remains without access to safely managed drinking water, defined by the WHO Joint Monitoring Program as water available on premises, available when needed, and free of microbial and chemical contaminants.[vi] More than 1 million deaths, due to diarrheal disease caused by waterborne illness, could be avoided annually with improved access to safe drinking water.[vii] In household and point-of-use settings, chlorination is also widely regarded as a potential low-cost solution for water treatment to prevent these deaths and improve microbial contamination of drinking water. However, although it is low-cost and relatively easy, using chlorine at the household level also suffers from the same potential for limited chlorine supply as it does in health care settings. Furthermore, uptake and sustained use is required for long term health outcomes but frequently lags over time for point-of-use chlorination.[viii]

Chlorine technologies and implementation

Electrolytic chlorine generators offer an attractive solution to addressing the key root causes of limited chlorine supply and stock outs in healthcare facilities. The ability to produce chlorine using simple inputs—water, salt, and electricity—at the push of a button make these devices well suited for low resource settings. When integrated into IPC and water treatment services, chlorine generators have the potential to significantly improve the quality of and trust in those services.

Passive chlorinators are a group of technologies that do not require electricity, automatically dose chlorine at or prior to the point of collection, and can provide drinking water that is chlorinated sufficiently to meet WHO standards for free chlorine residual and *E. coli*.[ix] Passive chlorination can adequately elevate the safety of drinking water supplies in contexts where drinking water is already available and free of chemical contaminants. In contrast to household water treatment, passive chlorination technologies reduce the burden on end users by providing chlorinated water directly at or before the point of water collection. These technologies have been installed in at least 16 countries, but recent analysis indicates that there is a global market of 2.32 billion people using contaminated water from sources that are compatible with passive chlorination.[x]

Though passive chlorinator and chlorine generators are technically distinct solutions, both can be implemented in healthcare settings and point-of-collection settings for chlorination needs, their potential for scale is inherently intertwined. Chlorine generators have the potential to meet the chlorine generation needs for passive chlorination systems.[ix] Research into the suitability and effectiveness of these solutions applies similar methodologies, and can be implemented in similar settings and contexts.

We see an opportunity to bring together interested stakeholders to create an open communication platform for sharing evidence, experiences, and approaches on how to stimulate the adoption of chlorine generators and how to advance the conversation around scaled implementation of passive chlorinators.

Purpose

The decentralized chlorine use Global Community of Practice (CoP) aims to be an international consortium of civil society organizations, private-sector companies, and individuals committed to advancing innovative chlorine generation technologies and service delivery models for disinfection and water treatment. The CoP will function as a global advocacy, networking, and learning alliance, aiming to stimulate collaborative and transparent discussion among partners on lessons learned, evidence gaps, and candid feedback on challenges faced through the deployment and use of on-site chlorine generators and passive chlorinators.

Collectively, CoP members will seek to contribute to two overarching health goals:

- Reduce the burden of water-borne diseases in low- and middle-income countries by supporting the use of chlorination technologies for decentralized water treatment in household and community-based water systems.
- Reduce the burden of hospital-acquired infections in low- and middle-income countries by supporting the use of chlorination technologies for improved infection prevention and control practices in healthcare facilities.

CoP benefits

We anticipate members, their beneficiaries, and the larger decentralized water treatment and IPC community to benefit in three distinct ways:

- **Improved awareness and technical knowledge** across all CoP members of electrolytic chlorine generator, passive dosing, and other related technologies and their potential to address service delivery gaps. By sharing implementation approaches, measurement indicators, user feedback, contextual challenges, and results to date, government and civil society members will be better informed when conducting ongoing or future pilots and introduction projects. Relevant manufacturers will similarly benefit by further understanding user needs, regulatory requirements, and how to more effectively deliver their products to consumers.
- **Strengthened collaboration** across organizations working to advance the adoption, integration, and use of on-site chlorine generators and passive dosing technologies and services. There are numerous organizations currently utilizing these technologies and many facing the same challenges and gaps. Through transparent

dialogue and virtual meetings, the CoP will enable its members to address these challenges collectively, deepening trust and confidence among its members.

- **Concerted advocacy and messaging** for stimulating innovation, policies, and accelerating market access of chlorine-related products. Championing the integration and adoption of electrolytic chlorine generators and passive dosing systems into health systems and markets will help strengthen the potential for sustained and continued future use of these products.

Participation and structure

Participation in the CoP is voluntary and open to civil society organizations, private-sector companies, and individuals who are involved, or interested, in the generation and use of chlorine products for disinfection and water treatment. We seek to create a diverse, inclusive, and equitable platform that fosters open and honest communication and encourages a broad range of views and backgrounds. The CoP is structured as a single convening body of members with up to four annual virtual meetings. Additional, topic specific side meetings may be held to allow for deeper dives into participants' topics, research, and discussion. Collaboration is key to the success of the CoP, and we welcome a diverse set of perspectives to be represented, including those from public, private, and civil society sectors.

One or more presentations on a technical topic or project may be included in each virtual meeting, and these may come from private-sector manufacturers or service providers, government representatives, or implementing organizations. The virtual meetings and calls will also serve as a venue for members to network with other members and to explore opportunities for collaboration and funding opportunities. Finally, presentations slides will be saved on the WASH in HCF COP website: https://www.washinhcf.org/cop/

Starting in June 2023, EOS International will join PATH as a co-secretariat of the CoP. We will revisit these roles in Q3 of 2024, following a year of joint CoP management. The secretariat will be responsible for organizing meetings and setting agendas, incorporating new members and maintaining the member list, serving as a central liaison for technical requests, and managing the overall direction of the CoP through consultation with partners. In addition, the co-secretariats will form and lead a steering committee, comprised of leading academic, civil society, and private sector actors. The steering committee will help shape the agenda and identify leading research/practitioners to feature in CoP sessions. Steering committee members will join on a voluntary basis and meet quarterly, a few weeks prior to each CoP session.

Any interested organization/corporation/individual may contact EOS International (Megan Lindmark megan.lindmark@eosintl.org) or PATH (Adam Drolet, adrolet@path.org) and provide a name, primary contact, and other contact information to be added to the CoP member list. A brief summary of current and/or planned activities related to on-site chlorine generators and specific interest in participating on the CoP should also be included.

CoP virtual meetings

Virtual meetings will be held quarterly and hosted by the co-secretariats. Roughly six weeks prior to the meeting, EOS International and PATH will host a meeting with the steering committee to identify leading researchers/project/practitioners to feature. A final agenda will be sent roughly a week prior to the meeting. A tentative schedule is shown in the table below.

Quarter	Date & time*
Q2 2023	Thursday, June 29th at 6 a.m. PST / 5 p.m. EAT / 7:30 p.m. IST
Q3 2023	Thursday, September 28th at 6 a.m. PST / 5 p.m. EAT / 7:30 p.m. IST
Q4 2023	Thursday, December 14th at 6 a.m. PST / 5 p.m. EAT / 7:30 p.m. IST
Q1 2024	Thursday, March 7th at 6 a.m. PST / 5 p.m. EAT / 7:30 p.m. IST

*To be confirmed. *Note*: EAT, East Africa Time; IST, India Standard Time; PST, Pacific Standard Time.

Several topics related to the adoption and use of on-site chlorine generators will be explored and discussed by the CoP. Areas of interest may include:

- **Technologies**: Product/technology agnostic, with manufacturers of products commercially available or in development invited to present on their technologies (specifications, benefits, evidence generated, etc.); contribute to discussions on challenges, barriers, and results to date; and collaboratively explore strategies to continue refining and improving product design and market introduction.
- **Regulatory, supply chain, and other supply-side issues**: In-country registration and regulatory authorization processes, manufacturing strategies, procurement processes, and supply chain, as well as post-sale support.
- **System integration, management models, and other demand-side issues**: Inclusive planning and policy integration, management models, demand/awareness generation, financing mechanisms and options, training strategies, service/infrastructure integration, monitoring and evaluation, operation and maintenance plans, and results dissemination approaches.
- **Evaluations on technical performance, usability, and operations**: Locations, use cases, implementation strategies, problem definition (including root causes of service gaps), beneficiaries and equity strategies, user acceptability, evaluation design and indicators, evidence to date and unanswered research gaps, and next steps.
- **Advocacy:** Advocacy to support for funding for electrolytic chlorine generator procurement and chlorine production, as well as monitoring and accountability for these activities via national and local health systems.

---

[i] World Health Organization (WHO). Health care-associated infections [fact sheet]. Geneva: WHO; 2016. Available at http://www.who.int/gpsc/country_work/gpsc_ccisc_fact_sheet_en.pdf.

[ii] Khan HA, Ahmad A, Mehboob R. Nosocomial infections and their control strategies. *Asian Pac J Trop Biomed*. 2015;5(7):509-514. doi:10.1016/j.apjtb.2015.05.001.

[iii] WHO. Maternal mortality [fact sheet]. Geneva: WHO; 2016. Available at http://www.who.int/mediacentre/factsheets/fs348/en/.

[iv] Oza S, Lawn J, Hogan D, et al. Neonatal cause-of-death estimates for the early and late neonatal periods for 194 countries: 2000–2013. Bulletin of the World Health Organization. 2015;93:19-28. Available at http://www.who.int/bulletin/volumes/93/1/14-139790/en/.

[v] Cronk R, Bartram J. Environmental conditions in health care facilities in low- and middle-income countries: Coverage and inequalities. *International Journal of Hygiene and Environmental Health*. 2018. https://doi.org/10.1016/j.ijheh.2018.01.004.

[vi] WHO and the United Nations Children's Fund. Progress on Household Drinking Water, Sanitation, and Hygiene 2000−2020:Five Years into the SDGs [fact sheet]. Geneva: WHO; 2021. Available at https://www.who.int/publications/i/item/9789240030848

[vii] WHO. The Global Health Observatory: Water, sanitation and hygiene: burden of disease. 2019. Available at https://www.who.int/data/gho/data/themes/topics/water-sanitation-and-hygiene-burden-of-disease

[viii] Crider YS, Tsuchiya M, Mukundwa M, Ray I, Pickering AJ. Adoption of Point-of-Use Chlorination for Household Drinking Water Treatment: A Systematic Review. Environmental Health Perspectives. 2023;131: 16001. https://doi.org/10.1289/EHP10839

[ix] Lindmark M, Cherukumilli K, Crider YS, Marcenac P, Lozier M, Voth-Gaeddert L, Lantagne DS, Mihelcic JR, Zhang QM, Just C, and Pickering AJ. Passive In-Line Chlorination for Drinking Water Disinfection: A Critical Review. Environmental Science & Technology 2022 56 (13), 9164-9181. https://doi.org/10.1021/acs.est.1c08580

[x] Cherukumilli K, Bain B, Chen Y, and Pickering AJ. Estimating the Global Target Market for Passive Chlorination. Environmental Science & Technology Letters. 2023. 10 (1), 105-110. https://doi.org/10.1021/acs.estlett.2c00781
