# Supplementary material for: Reinforcing Feedbacks for Sustainable Implementation of Rural Drinking-Water Treatment Technology
Source: ACS ES T Water. 2024 Mar 26;4(4):1763–74. doi: 10.1021/acsestwater.3c00779 (PMC11019543; doi:10.1021/acsestwater.3c00779)

SI Figure 1: *Conceptual visualization of key categories and factors in implementing passive chlorination and UV-C technology*

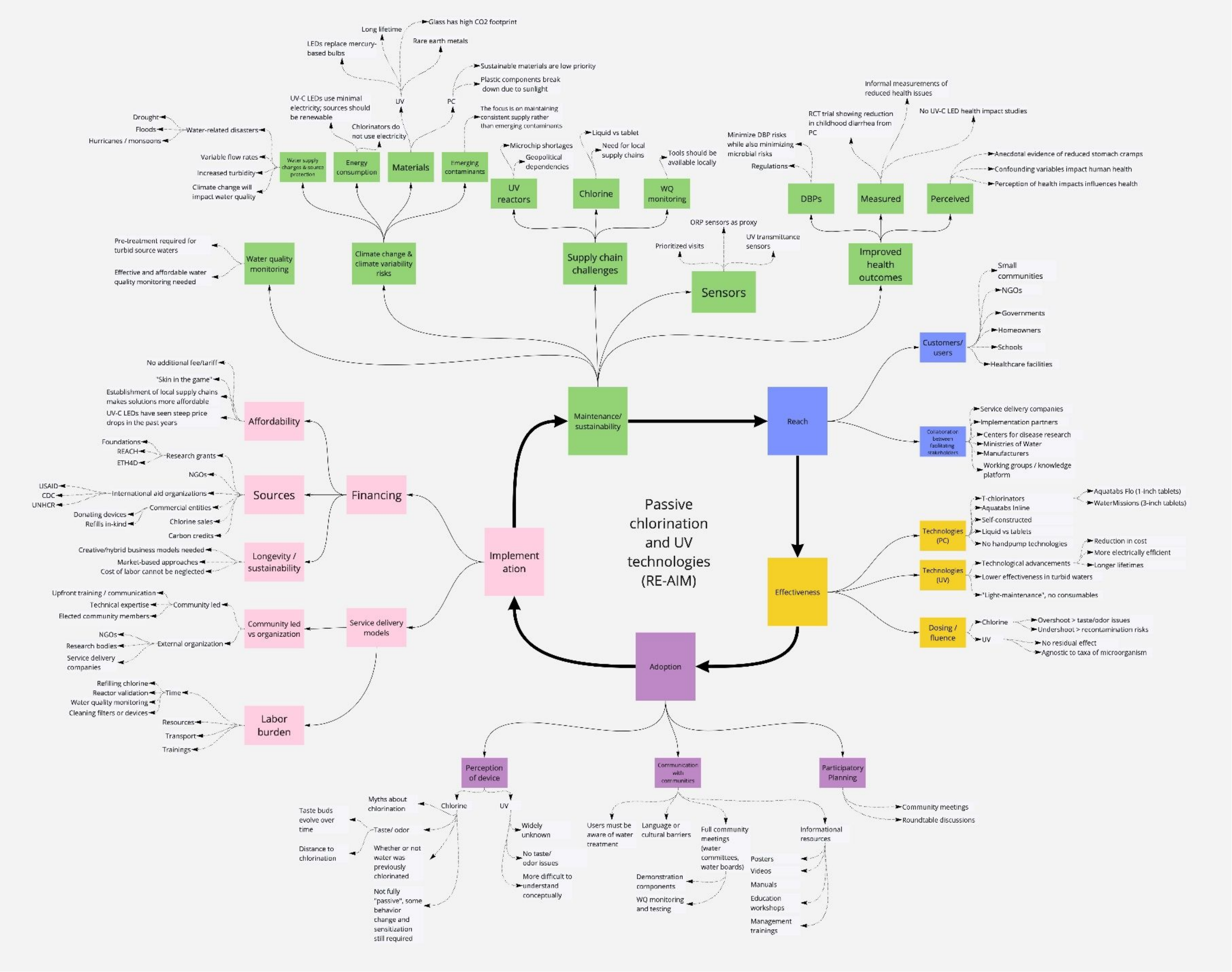

Supplement: Supplementary file 2 — ew3c00779_si_002.pdf [file ew3c00779_si_002.pdf]
